# Interventions for skin changes caused by nerve damage in leprosy

**DOI:** 10.1590/1516-3180.20131313T2

**Published:** 2013-06-01

**Authors:** Liv Merete Reinar, Louise Forsetlund, Arild Bjørndal, Diana Lockwood

## Abstract

**BACKGROUND:**

More than three million persons are disabled by leprosy worldwide. The main complication of sensory nerve damage is neuropathic ulceration, particularly of the feet. In this review we explored interventions that can prevent and treat secondary damage to skin and limbs.

**OBJECTIVE:**

To assess the effects of self-care, dressings and footwear in preventing and healing secondary damage to the skin in persons affected by leprosy.

**METHODS:**

*Search methods:* We searched the Cochrane Skin Group Specialised Register (April 2008), the Cochrane Central Register of Controlled Trials (The Cochrane Library Issue 1, 2008), MEDLINE (from 2003 to April 2008), EMBASE (from 2005 to April 2008), CINAHL (1982-2006) and LILACS (1982- April 2008) as well as online registers of ongoing trials (April 2008).

*Selection criteria:* Randomised controlled trials involving anyone with leprosy and damage to peripheral nerves treated with any measures designed to prevent damage with the aim of healing existing ulcers and preventing development of new ulcers.

*Data collection and analysis:* Two authors assessed trial quality and extracted data.

**MAIN RESULTS:**

Eight trials with a total of 557 participants were included. The quality of the trials was generally poor. The interventions and outcome measures were diverse. Although three studies that compared zinc tape to more traditional dressings found some benefit, none of these showed a statistically significant effect. One trial indicated that topical ketanserin had a better effect on wound healing than clioquinol cream or zinc paste, RR was 6.00 (95% CI 1.45 to 24.75). We did not combine the results of the two studies that compared topical phenytoin to saline dressing, but both studies found statistically significant effects in favour of phenytoin for healing of ulcer (SMD -2.34; 95% CI -3.30 to -1.39; and SMD -0.79; 95% CI -1.20 to 0.39). Canvas shoes were not much better than PVC-boots, and double rocker shoes did not promote healing much more than below-knee plasters.

**AUTHORS’ CONCLUSIONS:**

One study suggested that topical ketanserin is more effective than clioquinol cream or zinc paste. Topical phenytoin (two studies) may be more effective than saline dressing regarding ulcer healing. For the other dressings the results were equivocal. Canvas shoes were a little better than PVC-boots, but not significantly, and the effect of double rocker shoes compared to below-knee plasters was no different in promoting the healing of ulcers. No side effects were documented.

**This is the abstract of a Cochrane Review published in the Cochrane Database of Systematic Reviews** (CDSR) 2013, issue 1, Art. No. CD004833. DOI: 10.1002/14651858.CD004833.pub3 (http://onlinelibrary.wiley.com/doi/10.1002/14651858.CD004833.pub3/abstract). For full citation and authors details, see reference 1

**The full text is freely available, for Latin America and the Caribbean, from:** http://cochrane.bvsalud.org/cochrane/main.php?lib=COC&searchExp=Interventions%20and%20for%20and%20skin%20and%20changes%20and%20caused%20and%20by%20and%20nerve%20and%20damage%20and%20in%20and%20leprosy&lang=pt (this link may be temporary)
